# Fission yeast type 2 node proteins Blt1p and Gef2p cooperate to ensure timely completion of cytokinesis

**DOI:** 10.1186/s12860-018-0182-z

**Published:** 2019-01-24

**Authors:** Lois Kwon, Emma M. Magee, Alexis Crayton, John W. Goss

**Affiliations:** Department of Biological Sciences, Wellesley College, 106 Central Street, Wellesley, MA 02481 USA

**Keywords:** Septation initiation network, Cytokinesis, Fission yeast, Blt1p, Gef2p, Sid2p, Mob1p

## Abstract

**Background:**

The conserved NDR-family kinase Sid2p localizes to the contractile ring during fission yeast cytokinesis to promote ring constriction, septation, and completion of cell division. Previous studies have found that the Type 2 interphase node proteins Blt1p and Gef2p contribute to localization of Sid2p and its regulatory protein Mob1p at the division site. However, their relative contributions and whether they operate in the same or parallel pathways has been unclear. In this study, we quantify the respective roles of Blt1p and Gef2p in Sid2p/Mob1p recruitment and characterize the effect of single and double deletion mutants on contractile ring dynamics and completion of cell division.

**Results:**

Using quantitative confocal fluorescence microscopy, we measured Sid2p and Mob1p recruitment to the division site in *blt1∆*, *gef2∆*, and *blt1∆*/*gef2∆* mutant cells. We observed an equivalent decrease in Sid2p/Mob1p localization for both single and double mutants. Though assembly of the contractile ring is normal in these mutants, the reduction in Sid2p/Mob1p at the division site delayed the onset of contractile ring constriction and completion of division. We quantified localization of Blt1p and Gef2p at the medial cortex throughout the cell cycle and found that Blt1p localization to interphase nodes and the contractile ring is independent of Gef2p. However, Gef2p localization to the contractile ring is decreased in *blt1∆* mutants.

**Conclusions:**

Blt1p and Gef2p work in the same pathway, rather than in parallel, to localize the NDR-family kinase Sid2p and its regulatory partner Mob1p to the division site, thereby promoting timely completion of cell division. Future studies are necessary to understand how additional fission yeast cytokinesis proteins work with these Type 2 interphase node components to promote Sid2p/Mob1p recruitment.

**Electronic supplementary material:**

The online version of this article (10.1186/s12860-018-0182-z) contains supplementary material, which is available to authorized users.

## Background

The final stage of the eukaryotic cell cycle, cytokinesis, is driven by the assembly and constriction of an actin-myosin contractile ring [1, 2]. The proteins and mechanisms that regulate cytokinesis are evolutionarily conserved from fungi to humans [2–4]. In the fission yeast, *Schizosaccharomyces pombe*, entry into mitosis is regulated by the DYRK kinase Pom1p at the cell poles and the formation of two classes of interphase nodes at the medial cell cortex [5–8]. Type 1 interphase nodes form in early G2 and consist of the Cdr1p and Cdr2p kinases and the anillin-like protein Mid1p [6, 8, 9]. Type 2 interphase nodes are comprised of the putative RhoGef Gef2p, scaffolding proteins Blt1p and Nod1p, and the kinesin-like protein Klp8p, which migrate to the medial cortex from the disassembled contractile ring of the previous mitotic division [6, 8, 10–12]. Prior to the onset of mitosis, Type 1 nodes capture and combine with Type 2 nodes as they diffuse along the cortex [13]. When cells enter mitosis, the interphase nodes transition into cytokinesis nodes in preparation for contractile ring assembly as they recruit myosin-II heavy chain Myo2p and light chains Rlc1p and Cdc4p, F-BAR domain containing protein Cdc15p, formin Cdc12p, and IQGAP domain containing protein Rng2p [14–19]. Actin filaments polymerized by Cdc12p interact with myosin-II motors to condense cytokinesis nodes into the contractile ring through a search, capture, pull, and release mechanism [20–22]. Constriction of the fully formed contractile ring and synthesis of a cell wall septum by glucan synthases leads to separation of daughter cells and completion of cell division [23–25].

The onset of contractile ring assembly/constriction and septum formation in fission yeast is regulated by the Septation Initiation Network [26], which consists of a GTPase and kinase cascade [27–31]. This pathway shares homology with the *Saccharomyces cerevisiae* Mitotic Exit Network (MEN) and Hippo signaling pathway in *Drosophila melanogaster* and humans [31, 32]. Fission yeast SIN signaling proteins predominantly localize to the spindle pole body (SPB) during mitosis [30, 33]. Activation of the upstream Spg1p GTPase by Polo kinase Plo1p, as well as inactivation of the GTPase-activating proteins (GAP) Cdc16p and Byr4p, enables Spg1p to interact with Cdc7p kinase leading to asymmetric SIN signaling at one SPB [33–38]. Subsequent downstream activation of the SIN kinase Sid1p and its regulatory partner Cdc14p, in turn lead to activation of the NDR-family kinase Sid2p and its regulatory protein Mob1p [39–43]. Activated Sid2p/Mob1p moves from the SPB to the division site where it contributes to contractile ring compaction and constriction through phosphorylation of several substrates including the Cdc14-family phosphatase Clp1p, the morphogenesis Orb6 (MOR) pathway components Nak1p and Sog2p, and formin Cdc12p [26, 44–48]. Additionally, Sid2p kinase regulates accumulation of the (1,3)β-D-glucan synthase Bgs1p/Cps1p at the division site and timing of septum synthesis onset through the parallel MOR network [23, 24, 28, 49, 50]. In fission yeast, inactivation of Sid2p/Mob1p results in a failure to complete cytokinesis and division known as the “SIN phenotype” with elongated, multinucleated cells [40, 42, 51].

Previous studies found that Sid2p/Mob1p kinase complex recruitment to the division site prior to contractile ring constriction is decreased, but not eliminated, in cells lacking the Type 2 interphase node proteins Blt1p, Gef2p, or Nod1p, or upon deletion of the IQ-domain from Rng2p [12, 52, 53]. Additionally, Blt1p physically interacted with Sid2p and Mob1p, though it is not known if this interaction is direct or whether Blt1p interacts with one or both members of the Sid2p/Mob1p complex [52]. Decreased Sid2p/Mob1p localization led to delays in ring constriction, septum formation, and completion of division [52]. Of the Type 2 interphase node proteins implicated in kinase recruitment during cytokinesis, Blt1p localizes to the division site through interactions with Cdr2p and Cdc15p, while Gef2p and Nod1p are interdependent in their localization to the contractile ring [6, 11, 12]. This suggests that two independent mechanisms mediated by interphase node proteins might exist to recruit Sid2p/Mob1p to the division site, one via Blt1p and another through Gef2p and Nod1p, as redundant pathways to ensure proper kinase recruitment and progression of cytokinesis. In this study, we utilize quantitative fluorescence microscopy to determine the relative contributions of these putative Sid2p/Mob1p anchors and evaluate whether they work in the same pathway or as independent mechanisms. We quantify how the absence of both Type 2 interphase node proteins, Blt1p and Gef2p, impacts recruitment of Sid2p and Mob1p to the division site and how this double mutation affects contractile ring dynamics, septation, and completion of cell division.

## Results

### Characterization of blt1∆/gef2∆ double mutant growth

**Fig. 1:**
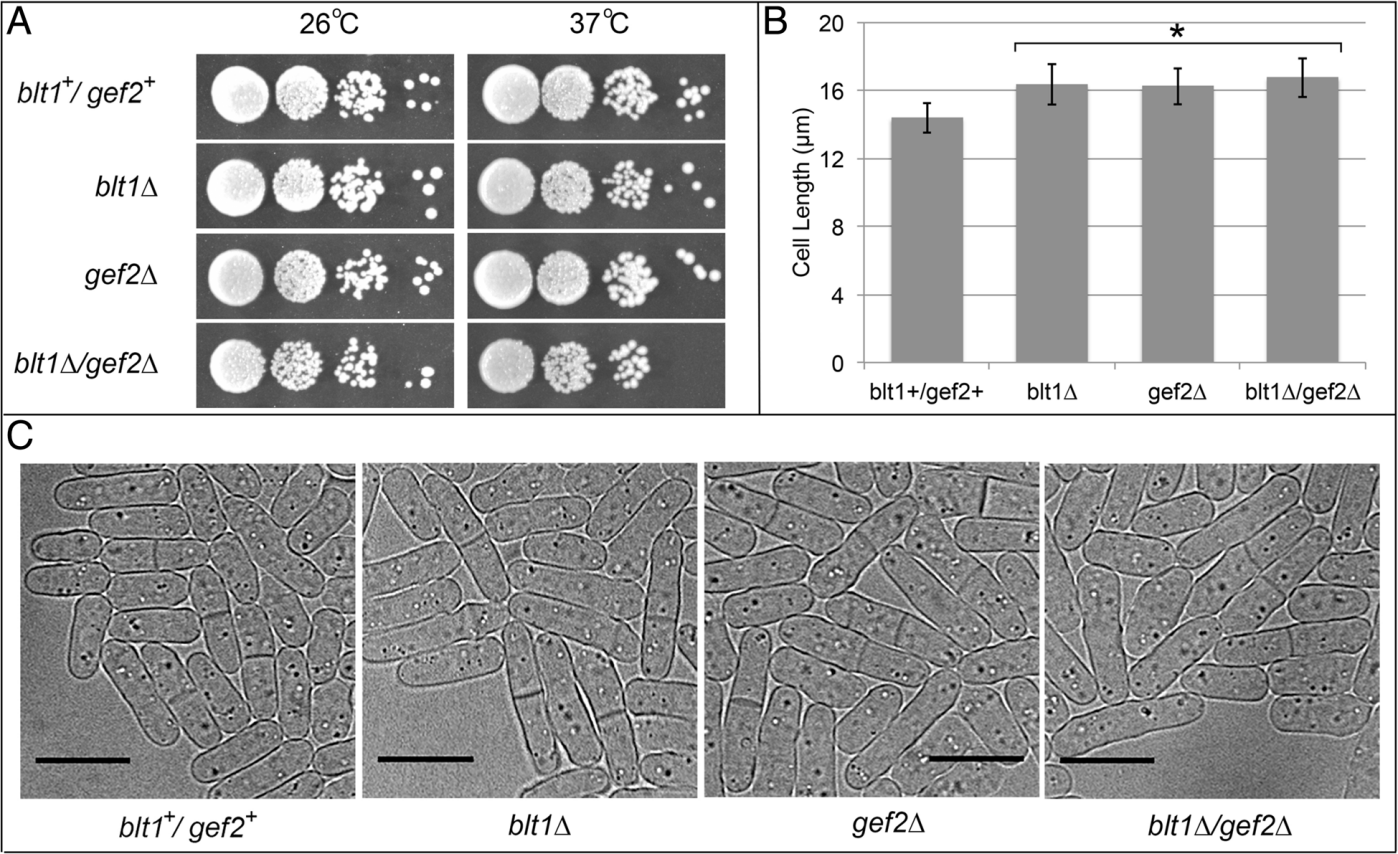
*blt1∆/gef2∆* mutant cells have decreased viability and are elongated at the completion of division. **a** Growth at 26 °C for 72 h or 37 °C for 48 h on YE5S agar plates with aliquots of 10-fold serial dilutions of wildtype cells (*blt1*^*+*^*/gef2*^*+*^) and *blt1∆*, *gef2∆*, and *blt1∆/gef2∆* mutant strains. **b** Histograms of the mean cell length ± 1 SD in septated cells prior to the initiation of cell wall indentation for wildtype (*blt1*^*+*^*/gef2*^*+*^), *blt1∆*, *gef2∆*, and *blt1∆/gef2∆* cells (*n* ≥ 25 cells for each condition). Asterisks indicate mean values of wildtype and mutant cells (*blt1∆*, *gef2∆*, *blt1∆*/*gef2∆*) that differed with *p* < 0.001. **c** White light transmission micrographs of wildtype (*blt1*^+^/*gef2*^+^), *blt1∆*, *gef2∆*, and *blt1∆*/*gef2∆* cells. Scale bar = 10 μm.

Independently, *blt1∆* or *gef2∆* mutations modestly decrease cellular viability, and *blt1∆* cells are elongated at the time of division, which is associated with delays in mitotic entry [6, 12, 52]. *blt1* has synthetic interactions with other interphase node constituents that lead to additive growth defects and an increase in the elongated cell length phenotype [6, 52]. This led us to investigate whether *blt1∆* and *gef2∆* have an additive effect on disrupting viability and cell size. Relative to *blt1*^*+*^/*gef2*^*+*^ wildtype cells grown at 37 °C, *blt1∆* and *gef2∆* each decreased (Fig. 1a). Growth of the *blt1∆*/*gef2∆* double mutant was further reduced (Fig. 1a), indicating a modest synthetic interaction between the two genes.

We analyzed the cell length of *blt1*^*+*^/*gef2*^*+*^ wildtype, *blt1∆*, *gef2∆*, and *blt1∆*/*gef2∆* double mutant cells to determine whether deletion of both genes has an additive effect on the cell length elongation phenotype. Consistent with previous reports, we found that wildtype cells were 14.4 ± 0.9 μm and *blt1∆* cells were 16.3 ± 1.2 μm immediately prior to completing division (Fig. 1b-c) [6]. *gef2∆* cells grew to 16.2 ± 1.0 μm and *blt1∆*/*gef2∆* double mutant cells to 16.7 ± 1.1 μm prior to dividing (Fig. 1b-c). The absence of an additive increase in cell size for the *blt1∆*/*gef2∆* double mutant suggests that the synthetic interaction between these two genes and growth defect is not related to mitotic entry.

### Blt1p and Gef2p work in tandem to localize Sid2p and Mob1p to the contractile ring

**Fig. 2:**
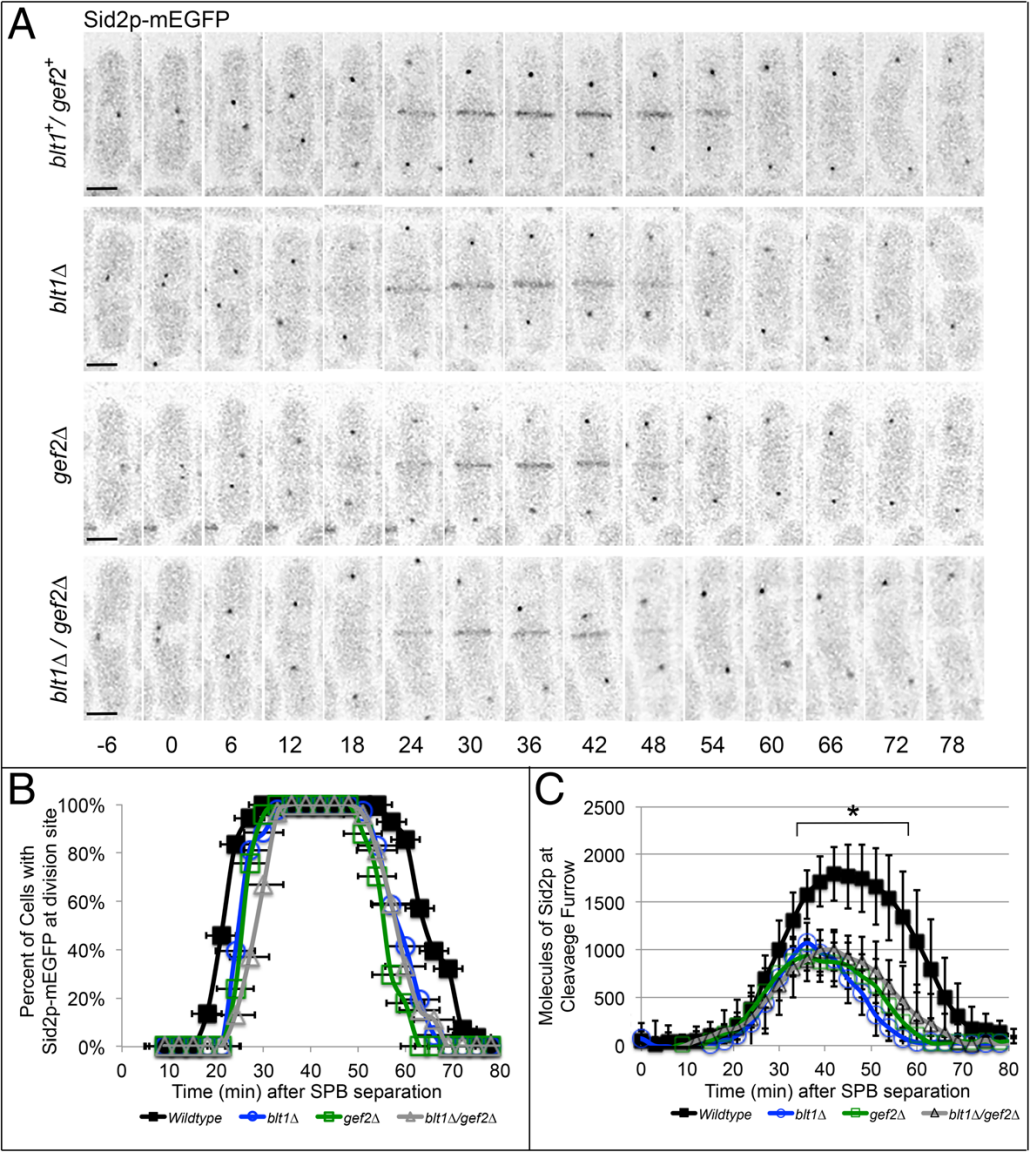
Blt1p and Gef2p function together in localization of Sid2p-mEGFP to the division site. Time shown in minutes; time zero represents SPB separation. **a** Time series of fluorescence micrographs at 6 min intervals in wild type (*blt1*^*+*^/*gef2*^*+*^, top row), *blt1∆* (second row), *gef2∆* (third row), or *blt1∆/gef2∆* (bottom row) cells expressing Sid2p-mEGFP (black). Scale bar = 3 μm. **b** Time course of Sid2p localization to the division site in wild type cells (black line, ; *n* = 37), *blt1∆* cells (blue line, ; *n* = 43), *gef2∆* cells (green line, ; *n* = 35); or *blt1∆*/*gef2∆* cells (gray line, Δ; *n* = 31). Error bars represent ±1 SD. **c** Time course of the mean number of molecules of Sid2p localized at the division site in wild type (black line ; n = 37), *blt1∆* (blue line ; n = 43), *gef2∆* (green line, ; n = 35), or *blt1∆/gef2∆* (gray line, Δ; n = 31) cells. Error bars represent ±1 SD. Asterisks indicate time points at which the mean values of wildtype and mutant cells (*blt1∆*, *gef2∆*, *blt1∆*/*gef2∆*) differed with *p* < 0.0001

Throughout most of the cell cycle Sid2p and Mob1p localize to SPBs, but in response to activation of SIN signaling during mitosis both move to the contractile ring [40, 42]. Quantitative analysis of *blt1*^*+*^/*gef2*^*+*^ wildtype cells by fluorescence confocal microscopy revealed that Sid2p-mEGFP appeared at the division site at + 23 ± 2 min (positive time indicates minutes after separation of SPB), concentrated to a maximum value of 1700 ± 260 molecules at + 39 min, and then gradually decreased until Sid2p-mEGFP was absent from the cell equator by + 65 ± 6 min (Fig. 2a-c). Consistent with previous findings, *blt1∆* or *gef2∆* cells showed reduced recruitment of Sid2p to the division site [12, 52]. Sid2p-mEGFP localized to the division site later (*blt1∆*: + 27 ± 4 min; *gef2∆*: 27 ± 3 min), reached a 41% lower peak concentration (*blt1∆*: 1000 ± 200 molecules at + 33 min; *gef2∆*: 1000 ± 210 molecules at + 36 min) and departed earlier (*blt1∆*: 54 ± 5 min; *gef2∆* 59 ± 4 min) in single mutant cells relative to wildtype cells (Fig. 2a-c). Recruitment of Sid2p-mEGFP to the cell equator was disrupted in *blt1∆/gef2∆* double mutant cells, but not to a greater extent than in *blt1∆* or *gef2∆* single mutants, arriving at 29 ± 3 min, departing at 61 ± 5 min, and reaching a peak concentration of 1000 ± 220 molecules at + 36 min (Fig. 2a-c; *p* > 0.05). The total number of Sid2p-mEGFP molecules within the cell during mitosis was the same between wildtype and mutant cells (Additional file 1 A).

**Fig. 3:**
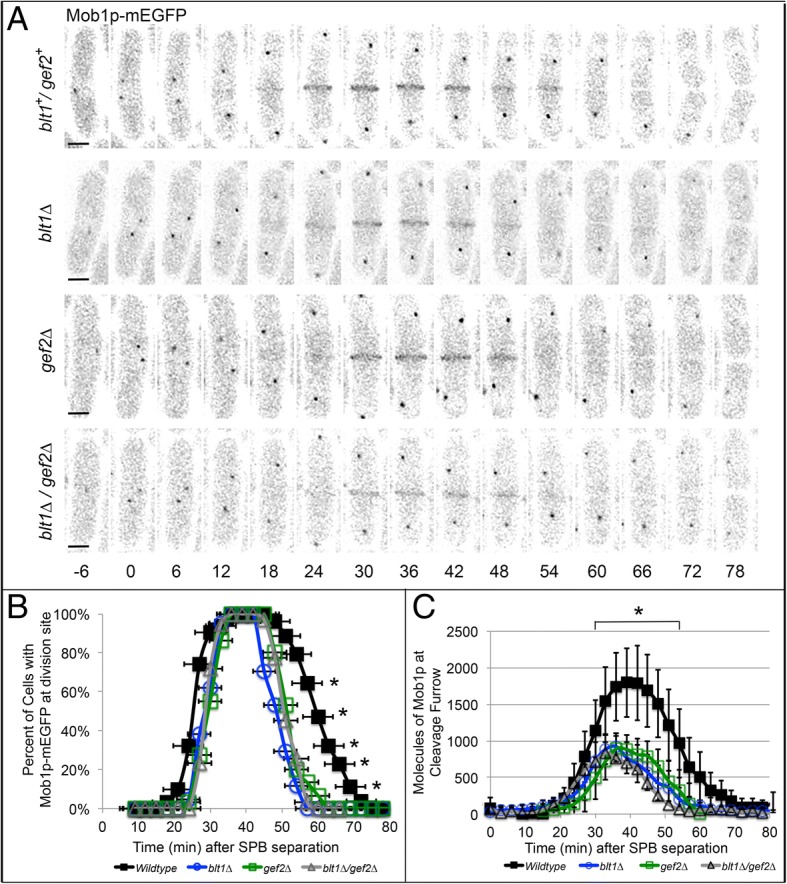
Blt1p and Gef2p function together in localization of Mob1p-mEGFP to the division site. Time shown in minutes; time zero represents SPB separation. **a** Time series of fluorescence micrographs at 6 min intervals in wild type (*blt1*^*+*^/*gef2*^*+*^, top row), *blt1∆* (second row), *gef2∆* (third row), or *blt1∆/gef2∆* (bottom row) cells expressing Mob1p-mEGFP (black). Scale bar = 3 μm. **b** Time course of Mob1p localization to the division site in wild type cells (black line, ; *n* = 66), *blt1∆* cells (blue line, ; *n* = 32), *gef2∆* cells (green line, ; n = 32); or *blt1∆*/*gef2∆* cells (gray line, Δ; *n* = 39). Error bars represent ±1 SD. **c** Time course of the mean number of molecules of Mob1p localized at the division site in wild type (black line, ; n = 66), *blt1∆* (blue line, ; n = 32), *gef2∆* (green line, ; n = 32), or *blt1∆/gef2∆* (gray line, Δ; n = 39) cells. Error bars represent ±1 SD. Asterisks indicate time points at which the mean values of wildtype and mutant cells (*blt1∆*, *gef2∆*, *blt1∆*/*gef2∆*) differed with *p* < 0.0001

Localization of Mob1p-mEGFP was similarly disrupted in *blt1∆*, *gef2∆*, and *blt1∆/gef2∆* mutant cells. Mob1p-mEGFP in wildtype cells localized to the division site at + 25 ± 4 min and concentrated to a maximum value of 1800 ± 370 molecules at + 39 min before leaving the cell equator by 63 ± 7 min (Fig. 3a-c). In strains lacking *blt1* or *gef2*, Mob1-mEGFP recruitment to the division site was delayed (*blt1∆*: 30 ± 3 min; *gef2∆*: 31 ± 3 min), the peak concentration was reduced by 50% (*blt1∆*: 900 ± 180 molecules at + 36 min; *gef2∆*: 900 ± 200 molecules at + 36 min), and it departed earlier (*blt1∆*: 55 ± 4 min; *gef2∆*: 58 ± 6 min) relative to localization in wildtype cells (Fig. 3a-c). Double mutants lacking both *blt1* and *gef2* disrupted Mob1p-mEGFP localization to the same extent as single mutant cells, arriving at the division site at 28 ± 3 min, departing at 57 ± 3 min, and reaching a peak concentration of 900 ± 220 molecules at + 36 min (Fig. 3a-c; *p* > 0.05). The total number of Mob1p-mEGFP molecules within the cell during mitosis was consistent between wildtype and mutant cells (Additional file 1 B). The combined loss of Blt1p and Gef2p does not lead to an additive reduction in recruitment of Sid2p-mEGFP and Mob1p-mEGFP from the SPB to the division site during cytokinesis. This suggests that Blt1p and Gef2p work together, rather than independently, to localize the Sid2p/Mob1p complex to the division site during cytokinesis.

### Blt1p and Gef2p work together to ensure timely constriction of the contractile ring

**Fig. 4:**
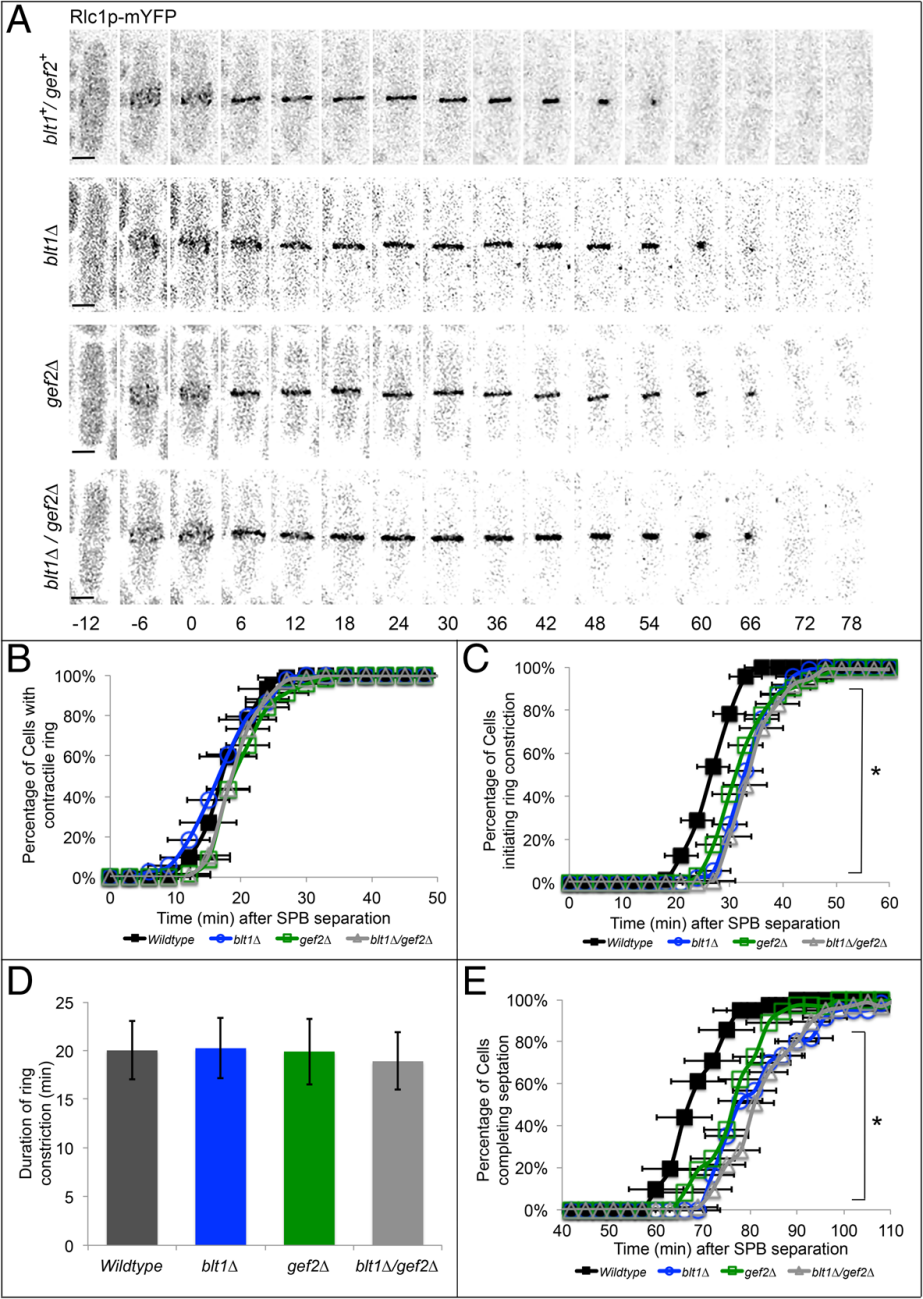
Onset of contractile ring constriction and septation are delayed in *blt1∆*, *gef2∆*, and *blt1∆/gef2∆* mutants. Time shown in minutes; time zero represents SPB separation. **a** Time series of fluorescence micrographs at 6 min intervals in wild type (*blt1*^*+*^*/gef2*^*+*^, top row), *blt1∆* (second row), *gef2∆* (third row), or *blt1∆/gef2∆* (bottom row) cells expressing Rlc1p-mYFP (black). Scale bar = 3 μm. **b** Time course of the completion of Rlc1p nodes coalescence into the contractile ring in wildtype (black line, ; *n* = 92), *blt1∆* cells (blue line, ; *n* = 107), *gef2∆* cells (green line, ; *n* = 69) or *blt1∆*/*gef2∆* cells (gray line, Δ; *n* = 121). Error bars represent ±1 SD. **c** Time course of the onset of contractile ring constriction in wildtype cells (black line, ; *n* = 87), *blt1∆* cells (blue line, ; *n* = 96), *gef2∆* cells (green line, ; *n* = 63) or *blt1∆*/*gef2∆* cells (gray line, Δ; *n* = 112). Asterisks indicate time points at which the mean values of wildtype and mutant cells (*blt1∆*, *gef2∆*, *blt1∆*/*gef2∆*) differed with *p* < 0.0001. Error bars represent ±1 SD. **d** Mean duration of time to completion of contractile ring constriction in wildtype (wt, black) and mutant cells (*blt1∆*, blue; *gef2∆*, green; *blt1∆*/*gef2∆*, gray). Error bars represent ±1 SD. **e** Time course of septation completion resulting in two independent cells in wildtype (black line, ; *n* = 41), *blt1∆* (blue line, ; *n* = 60), *gef2∆* (green line, ; *n* = 37); or *blt1∆*/*gef2∆* cells (gray line, Δ; *n* = 75). Error bars represent ±1 SD. Asterisks indicate time points at which the mean values of wildtype and mutant cells (*blt1∆*, *gef2∆*, *blt1∆*/*gef2∆*) differed with *p* < 0.0001

Because decreased recruitment of Sid2p/Mob1p to the division site delays the onset of contractile ring constriction, we investigated contractile ring dynamics in *blt1*^*+*^/*gef2*^*+*^ wildtype, *blt1∆*, *gef2∆*, and *blt1∆*/*gef2∆* cells using confocal fluorescence microscopy [52, 53]. Recruitment of the type II myosin regulatory light chain, Rlc1p, to nodes and the coalescence of those nodes into the contractile ring occurred at the same times in wildtype, *blt1∆*, *gef2∆*, and *blt1∆*/*gef2∆* cells, indicating normal assembly of the contractile ring (Additional file 2 A; Fig. 4a-b; *p* > 0.05). Additionally, the timing of other phases of mitosis (anaphase A, anaphase B, and telophase) were normal in *blt1∆*, *gef2∆*, and *blt1∆*/*gef2∆* cells (Additional file 2 B-D; *p* > 0.05).

Despite normal progression through early mitosis and assembly of the contractile ring, delays occurred in initiation of ring constriction and completion of division. In wildtype cells, constriction of the contractile ring was initiated at 26 ± 4 min, whereas constriction onset was delayed 10 min in mutant cells (*blt1∆*: 36 ± 4 min; *gef2∆*: 35 ± 6 min; *blt1∆/gef2∆*: 35 ± 4 min) (Fig. 4c). The duration of ring constriction was the same between wildtype (20 ± 3 min) and mutant cells (*blt1∆*: 20 ± 3 min; *gef2∆*: 19 ± 4 min; *blt1∆*/*gef2∆*: 18 ± 3 min) (Fig. 4d; *p* > 0.05), indicating no changes in constriction rate. The completion of septation, resulting in two independent daughter cells, occurred in wildtype cells at 69 ± 6 min, but was delayed by approximately 10 min in *blt1∆* cells (81 ± 8 min), *gef2∆* cells (78 ± 7 min), or *blt1∆*/*gef2∆* cells (81 ± 7 min) (Fig. 4e). This finding is consistent with previous reports that reduced recruitment and retention of Sid2p/Mob1p at the division site in *blt1∆* mutants delays the onset of contractile ring constriction and completion of division [29, 52]. Moreover, the loss of both *blt1* and *gef2* does not have an additive effect on these delays.

### Blt1p is required for Gef2p retention at the contractile ring during division

**Fig. 5:**
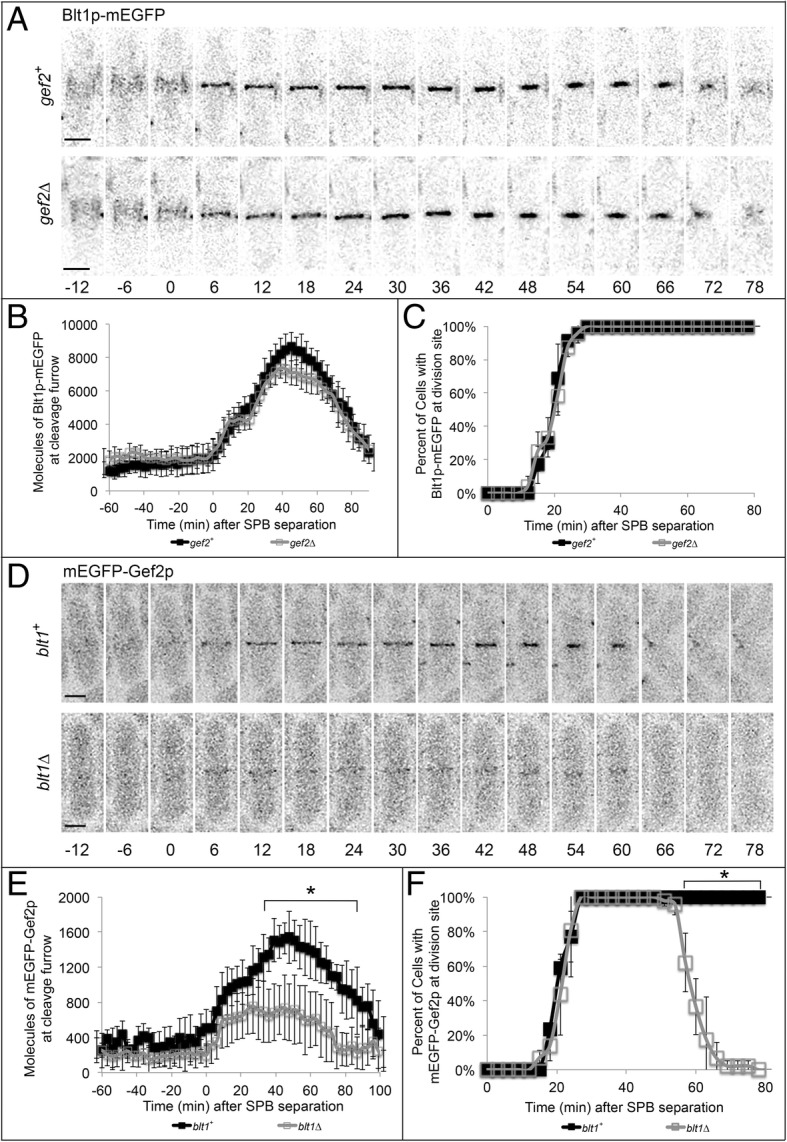
mEGFP-Gef2p localization to the division site is disrupted in *blt1∆* mutants. Time shown in minutes; time zero represents SPB separation. **a** Time series of fluorescence micrographs at 6 min intervals in wild type (*gef2*^*+*^, top row) and *gef2∆* (bottom row) cells expressing Blt1p-mEGFP (black). Scale bar = 3 μm. **b** Time course of the mean number of molecules of Blt1p localized at the division plane in wild type cells (black line, ; *n* = 30) and *gef2∆* cells (grey line, ; *n* = 24). Error bars represent ±1 SD. **c** Time course of Blt1p localization to the division site in wild type cells (black line, ; *n* = 23) and *gef2∆* cells (grey line, ; n = 24). Error bars represent ±1 SD. **d** Time series of fluorescence micrographs taken at 6 min intervals in wildtype (*blt1*^*+*^, top row) and *blt1∆* (bottom row) cells expressing mEGFP-Gef2p (black). Scale bar = 3 μm. **e** Time course of the mean number of molecules of Gef2p localized at the division plane in wild type cells (black line, ; *n* = 22) and *blt1∆* cells (grey line, ; *n* = 39). Error bars represent ±1 SD. **f** Time course of Gef2p localization to the division site in wild type cells (black line, ; n = 22) and *blt1∆* cells (grey line, ; *n* = 37). Error bars represent ±1 SD. Asterisk indicates range at which the mean values of wildtype and *blt1∆* mutant cells differ with *p* < 0.001.

Blt1p and Gef2p directly interact in interphase nodes and the contractile ring [6, 11, 54]. We used time-lapse fluorescence confocal microscopy to determine the timing of this interaction and quantify localization of Blt1p and Gef2p at nodes and the contractile ring throughout the cell cycle. During interphase, the concentration of Blt1p-mEGFP in type 2 nodes gradually increases from 900 ± 360 molecules at − 80 min (negative time indicates minutes prior to SPB separation) to 2200 ± 930 molecules at + 3 min, which marks the beginning of contractile ring formation (Fig. 5a-b). Blt1p-labeled cytokinesis nodes then coalesce to form the contractile ring, and additional Blt1p is recruited until it reaches a peak value of 8500 ± 870 molecules at + 42 min (Fig. 5a-b). Following completion of contractile ring constriction, Blt1p is retained at the division site and slowly departs during septation (from 6500 ± 1020 at + 66 to 2000 ± 830 molecules at + 90 min) as it migrates to the medial region of the cell to join type 2 nodes (Fig. 5a-c). Deletion of *gef2* did not significantly disrupt recruitment of Blt1p-mEGFP to type 2 nodes during interphase (900 ± 500 molecules at − 80 min to 2300 ± 750 molecules at + 3 min), peak value during ring constriction (7600 ± 820 at + 42 min), or departure from the division site (Fig. 5a-c; *p* > 0.05). This indicates that Gef2p does not influence Blt1p localization to interphase nodes, cytokinesis nodes, the contractile ring, or the division site.

Gef2p-mEGFP is recruited to type II nodes early in interphase, increasing from 300 ± 150 molecules at − 80 min to 500 ± 190 molecules at + 3 min (Fig. 5d-e). As Gef2p-mEGFP labeled cytokinesis nodes coalesce into the contractile ring, the concentration at the division site increases to a maximum value of 1500 ± 300 molecules at + 48 min (Fig. 5d-e). After completion of ring constriction, Gef2p remains at the division site and slowly leaves following septation (1200 ± 230 molecules at + 66 min to 700 ± 220 molecules at + 90 min) to join to medial type 2 nodes (Fig. 5d-f). Deletion of *blt1* slightly reduced recruitment of Gef2p-mEGFP to type 2 interphase nodes (200 ± 100 molecules at − 80 min to 300 ± 210 molecules at + 3 min) (Figs. 5d-e). As nodes coalesced into the contractile ring, the amount of Gef2p at the division site was reduced by 53% in *blt1∆* cells (700 ± 370 molecules at + 45 min) (Fig. 5d-e). In contrast to wildtype cells, the remaining Gef2p was not retained at the division site following ring constriction and prior to completion of division in *blt1∆* mutants (500 ± 360 molecules at + 66 min to 200 ± 180 molecules at + 90 min) (Fig. 5d-f). This suggests that Blt1p contributes to Gef2p recruitment to type 2 interphase nodes and is important for Gef2p retention at the contractile ring and during septation.

## Discussion

The Septation Initiation Network [26] is an essential signaling pathway, homologous to the budding yeast MEN and mammalian Hippo signaling pathways, that contributes to contractile ring compaction/constriction and the initiation of septation in fission yeast [29, 30, 32]. Translocation of the NDR-family kinase Sid2p and its regulatory subunit Mob1p to the division site communicates SIN signaling to the contractile ring [40, 42, 43]. When localized to the division plane, the Sid2p kinase promotes contractile ring compaction and activation of primary septum synthesis at the division site, which contributes to contractile ring constriction, septum formation, and completion of cell division [23–25, 28, 44–46, 49]. Fission yeast Type 2 interphase node proteins Blt1p, Gef2p, and Nod1p as well as the IQ-domain of Rng2p have been implicated in anchoring the Sid2p/Mob1p complex at the division site following its dissociation from the SPB, which in turn enables its interactions with its downstream substrates [12, 52, 53]. Decreased Sid2p/Mob1p localization to the contractile ring contributes to delayed ring constriction, septation, and completion of division [52].

In this study, we utilized quantitative fluorescence confocal microscopy to determine whether the Type 2 interphase node proteins, Blt1p and Gef2p, operate in the same or parallel pathways for recruiting Sid2p and Mob1p to the division site. Consistent with previous studies, Sid2p and Mob1p recruitment and retention at the division site was significantly reduced upon deletion of *blt1* or *gef2* [12, 52, 55]. Deletion of both *blt1* and *gef2* did not lead to an additional disruption of Sid2p or Mob1p localization relative to the single mutant deletion phenotype. Likewise, *blt1∆*/*gef2∆* double mutants did not have an additive effect on delaying contractile ring constriction, septation, or completion of division. This suggests that Blt1p and Gef2p operate in the same pathway for recruitment of Sid2p and Mob1p to the division site thereby working together to ensure timely completion of division.

The onset of ring constriction in *blt1∆*, *gef2∆*, or *blt1∆*/*gef2∆* mutants at approximately + 36 min correlated with timing of the peak concentration of ~ 1000 molecules of Sid2p/Mob1p at the division site. Wildtype cells reached this level of Sid2p/Mob1p recruitment at approximately + 27 min, which is consistent with their initiation of contractile ring constriction at + 26 ± 4 min. This correlation between a threshold for Sid2p/Mob1p recruitment and the onset of ring constriction is consistent with previous findings for wildtype and *blt1∆* cells [52]. Since *blt1∆*/*gef2∆* cells do not further decrease Sid2p/Mob1p localization to the division site or have an additive increase in the delay of constriction onset and septation, the mild synthetic growth defect observed for double mutants at high temperatures in the growth assay is likely the result of disrupted interactions with other interphase or cytokinesis proteins that interfere with proper division. These other putative interactions and their contributions to successful cell division should be investigated in future studies.

In the absence of both Blt1p and Gef2p, a reduced amount of Sid2p and Mob1p is still recruited to the contractile ring, which enables the delayed completion of division. Future studies are needed to assess the relative contributions of other components (Type 2 interphase node protein Nod1p and IQGAP protein Rng2p) implicated in Sid2p/Mob1p recruitment [12, 53]. Since Nod1p complexes with Blt1p and Gef2p in Type 2 nodes and localization of Nod1p to nodes and the contractile ring is dependent upon Gef2p [11, 12], it is most likely involved in Blt1p/Gef2p-mediated recruitment of Sid2p and Mob1p. However, Rng2p does not appear in interphase nodes and is recruited to the contractile ring during mitosis [53, 56, 57], suggesting that an independent Rng2p-mediated pathway might be responsible for the observed Sid2p/Mob1p localization to the division site in the absence of both Blt1p and Gef2p.

Since these type 2 node proteins work together in recruiting Sid2p and Mob1p to the division site, we next quantified the timing of Blt1p and Gef2p localization throughout the cell cycle to determine the hierarchy of their interaction. We found that Blt1p is recruited to type 2 interphase nodes independently of Gef2p, likely mediated through its interaction with Cdr2p [6, 10]. In the absence of Gef2p, there was no significant reduction of Blt1p in interphase nodes at the division site, during node coalescence into the contractile ring during mitosis, or during ring constriction and cytokinesis. On the other hand, Gef2p recruitment to interphase nodes was reduced, but not abolished, in *blt1∆* mutants. Previously characterized interactions with other interphase node proteins, Nod1p and Mid1p, are likely to compensate for Gef2p recruitment and retention at nodes in the absence of Blt1p [10–12, 54]. *blt1∆* mutants displayed a significant reduction in Gef2p at the contractile ring during mitosis and cytokinesis, suggesting that Blt1p is important for Gef2p retention at the division site following Mid1p departure from the contractile ring [11, 12, 14, 49]. Our quantitative confocal microscopic analysis of the total molecules of Blt1p and Gef2p recruited to nodes and the newly formed contractile ring was consistent with recent studies utilizing superresolution microscopy [13]. Taken together, this suggests that Blt1p promotes localization of Gef2p at the contractile ring during cytokinesis, which in turn contributes to recruitment and retention of Sid2p and Mob1p at the division site. Reduced Sid2p/Mob1p recruitment to the contractile ring in the absence of either Blt1p or Gef2p delays the onset of ring constriction, septation, and completion of division. The human homologues of fission yeast Sid2p and Mob1p (LATS1 kinase and MOB1 regulatory subunit, respectively) localize to the division site during mitosis and cytokinesis [58–60]. These findings suggest that future studies in mammalian cells could focus on structural homologues of Blt1p or Gef2p that contribute to LATS1 and MOB1 recruitment to the division site and investigate their contribution to mammalian cell division.

## Conclusions

In conclusion, this study finds that the Type 2 interphase node protein Blt1p promotes retention of the putative RhoGEF, Gef2p, at the contractile ring during cytokinesis, which in turn contributes to recruitment and retention of the NDR-family kinase, Sid2p, and its regulatory protein, Mob1p, at the division site. Proper Sid2p/Mob1p localization through Blt1p/Gef2p is necessary for the timely onset of contractile ring constriction, septation, and completion of cytokinesis.

## Methods

### Strains and growth conditions

Fission yeast strains used in this study are listed in Supplemental Table 1. Fluorescent protein tags in strains were generated using PCR-based targeting protocols at the endogenous chromosomal locus, enabling expression under the control of the endogenous promoter [61]. Cells were cultured in YE5S medium at 25 °C in mid-log phase (OD_595_ < 0.6) for 36 h prior to microscopic studies.

**Table 1:** Strain list

Strains	Genotype	Source/Reference
JG1–1	*h- blt1∆::natMX6 ade6-M210 leu1–32 ura4-D18*	Goss et al., 2014 [52]
JG3–4	*h- blt1-mEGFP-kanMX6 sad1-RFP-kanMX6 ade6-M210 leu1–32 ura4-D18*	Goss et al., 2014 [52]
JG114-2A	*h + gef2∆::kanMX6 mob1-mEGFP-kanMX6*	This study: JW1826 x CM26
JG115-5B	*h + gef2∆::kanMX6 sid2-mEGFP-kanMX6*	This study: JW1826 x CM77
JG121-4A	*h + gef2∆::kanMX6 rlc1-mYFP-kanMX6 sad1-CFP-kanMX6 ade6-M210 leu1–32 ura4-D18*	This study: JW991 x JW1826
JG124-1A	*h + gef2∆::kanMX6 blt1-mEGFP-kanMX6 sad1-RFP-kanMX6 ade6-M210 leu1–32 ura4-D18*	This study: JG3–4 x JW1826
JG131–1B	*h- blt1∆::natMX6 gef2∆::kanMX6 Sid2-mEGFP-kanMX6 ade6-M210 leu1–32 ura4-D18*	This study: JG1–1 x JG115
JG148-9B	*h + blt1∆::kanMX6 mob1-mEGFP-kanMX6 ade6-M210 leu1–32 ura4-D18*	This study: JM206 x CM77
JG153-7A	*h + blt1∆::kanMX6 sid2-mEGFP-kanMX6 ade6-M210 leu1–32 ura4-D18*	This study: JM206 x CM26
JG164-6C	*h + blt1∆::kanMX6 gef2∆::kanMX6 mob1-mEGFP-kanMX6 ade6-M210 leu1–32 ura4-D18*	This study: JG148 x JG114
JG166-3B	*h + blt1∆::kanMX6 gef2∆::kanMX6 ade6-M210 leu1–32 ura4-D18*	This study: JM206 x JW1826
JG171-3C	*h + blt1∆::natMX6 rlc1-mYFP-kanMX6 sad1-CFP-kanMX6 ade6-M210 leu1–32 ura4-D18*	This study: JG1 x JW991
JG172-3A	*h + blt1∆::natMX6 gef2∆::kanMX6 rlc1-mYFP-kanMX6 sad1-CFP-kanMX6 ade6-M210 leu1–32 ura4-D18*	This study: JG121 x JG172
CB97	*h- arp3-mEGFP-kanMX6 ade6-M210 leu1–32 ura4-D18*	TD Pollard
CM26–3	*h- sid2-mEGFP-kanMX6 ade6-M210 leu1–32 ura4-D18*	TD Pollard
CM77	*h- mob1-mEGFP-kanMX6 ade6-M210 leu1–32 ura4-D18*	TD Pollard
JM206	*h + blt1∆::kanMX6 ade6-M210 leu1–32 ura4-D18*	Moseley et al., 2009 [6]
JQ1109	*h + kanMX6-P*_*myo2*_^*+*^ *-mEGFP-myo2 ade6-M210 leu1–32 ura4-D18*	TD Pollard
JQ1114	*h- ain1-mEGFP-kanMX6 ade6-M210 leu1–32 ura4-D18*	TD Pollard
JW81	*h- ade6-M210 leu1–32 ura4-D18*	Wu et al., 2003 [14]
JW729	*h + ade6-M210 leu1–32 ura4-D18*	Wu et al., 2003 [14]
JW991	*h + rlc1-mYFP-kanMX6 sad1-CFP-kanMX6 ade6-M210 leu1–32 ura4-D18*	Wu et al., 2003 [14]
JW1826	*h + gef2∆::kanMX6 ade6-M210 leu1–32 ura4-D18*	Ye et al., 2012 [54]
JW4227	*h- kanMX6-P*_*gef2*_^*+*^ *-mEGFP-4Gly-gef2 sad1-tdTomato-natMX6 ade6-M210 leu1–32 ura4-D18*	Ye et al., 2012 [54]
JW4237	*h- blt1∆::kanMX6 kanMX6-P*_*gef2*_^*+*^ *-mEGFP-4Gly-gef2 sad1-tdTomato-natMX6 ade6-M210 leu1–32 ura4-D18*	Ye et al., 2012 [54]
TP226	*h- arpc5-mEGFP-kanMX6 ade6-M210 leu1–32 ura4-D18*	TD Pollard
TP237	*h + acp2-mEGFP-kanMX6 ade6-M210 leu1–32 ura4-D18*	TD Pollard
TP347	*h- aim1-mEGFP-kanMX6 ade6-M210 leu1–32 ura4-D18*	TD Pollard

### Microscopy, data analysis, and counting numbers of molecules

Cells for microscopic analysis were centrifuged at 500 g and washed three times with EMM5S medium. Cells were plated on a 25% gelatin pad prepared with EMM5S medium supplemented with 0.1 mM n-propyl-gallate. Fluorescence microscopy was performed at 25 °C using a Leica DMI 6000 CS microscope with a Leica TCS SP5 II confocal scan unit (Leica Microsystems, Wetzlar, Germany) and a 100x/NA 1.44 Plan Apo lens (Leica). An argon laser (488 nm at 20% laser power output) or helium-neon laser (594 nm at 33% laser power output) were used for exciting fluorophores. Stacks of 10 Z-slices at 1.0 μm intervals were acquired every 3 min at 100 Hz for 1–3 h. Data were collected from multiple cells in multiple experiments for each condition evaluated.

Sum projections for confocal stack images acquired from microscopic experiments were generated and analyzed using ImageJ software and macros (National Institutes of Health, Bethesda, MD). We used a two-tailed Student’s *t*-test to determine the statistical significance in the timing of fluorescently-tagged protein localization to the division site, contractile ring constriction onset and completion, and timing of septation in wildtype and mutant cells. We used a log-rank test (MedCalc Software, Ostend, Belgium) to calculate whole outcome plots for comparison of wildtype and mutant strains and a *p*-value for each pair of curves.

Confocal stacks of images acquired from cells expressing fluorescent tags were taken under identical conditions given above. Sum stacks for all sections with fluorescent signals were corrected for camera noise and uneven illumination [62]. Cells were imaged without an excitation light source to correct for camera noise. Uneven illumination corrections were made using a confocal stack of images from a uniform field of AlexaFluor-488 dye on a coverslip. The fluorescence intensity of wildtype cells was subtracted from images to account for autofluorescence. Reference cells were imaged in parallel with experimental samples to subtract the effects of acquisition photobleaching from our measurements. Cellular and local concentrations and numbers of molecules were determined by comparison of mEGFP fluorescence between experimental cells and a calibration curve consisting of strains expressing fluorescently-tagged proteins with known cellular concentrations [52, 62, 63] (Additional file 3).

### Cellular viability and growth assay

*S. pombe* cells were cultured for 24 h on a rotary wheel in YE5S liquid medium at 25 °C, diluted 1:100, and cultured an additional 16 h at 25 °C. Equal numbers of cells were taken from cultures in exponential growth phase (OD_595_ < 0.6) and serial diluted 10-fold four times. 5uL aliquots from each dilution were spotted onto YE5S agar plates and incubated at 25 °C, 30 °C or 36 °C for 36–72 h. Plates were imaged with transmitted light by a GelDoc XR imager (BioRad, Hercules, CA).

## Additional files

Additional file 1: Cellular concentrations of Sid2p-mEGFP and Mob1p-mEGFP are constant between wildtype and mutant strains during mitosis. (ZIP 696 kb)

Additional file 2: Rlc1p-mYFP recruitment to nodes and initiation of anaphase and telophase are normal in *blt1∆*, *gef2∆*, and *blt1∆/gef2∆* mutants. (ZIP 1277 kb)

Additional file 3: Standard curve with measurement of mean molecules per cell by fluorescence microscopy. (ZIP 507 kb)

## Abbreviations

GAP: GTPase Activating Protein
mEGFP: Monomeric Enhanced Green Fluorescent Protein
MEN: Mitotic Exit Network
MOR: Morphogenesis Orb6
SIN: Septation Initiation Network
SPB: Spindle Pole Body
