# Supplementary material for: Fission yeast type 2 node proteins Blt1p and Gef2p cooperate to ensure timely completion of cytokinesis
Source: BMC Mol Cell Biol. 2019 Jan 24;20:1. doi: 10.1186/s12860-018-0182-z (PMC6446504; doi:10.1186/s12860-018-0182-z)
Supplement: Supplementary file 1 — Cellular concentrations of Sid2p-mEGFP and Mob1p-mEGFP are constant between wildtype and mutant strains during mitosis. (ZIP 696 kb) [file 12860_2018_182_MOESM1_ESM.zip › Additional File 1.docx]

**Additional File 1**: **Cellular concentrations of Sid2p-mEGFP and Mob1p-mEGFP are constant between wildtype and mutant strains during mitosis.**

Time shown in minutes; time zero represents SPB separation. Time course of mean number ± 1 SD of (A) Sid2p-mEGFP molecules and (B) Mob1-mEGFP molecules in wildtype (black line, ◼; n = 20), *blt1Δ* cells (blue line, 🞆; n = 20), *gef2Δ* cells (green line, ☐; n = 20) or *blt1Δ*/*gef2Δ* cells (gray line, Δ; n = 20). Error bars represent ± 1 SD. *p-value* > 0.1.
