# Supplementary figures and images for: Fission yeast type 2 node proteins Blt1p and Gef2p cooperate to ensure timely completion of cytokinesis
Source: BMC Mol Cell Biol. 2019 Jan 24;20:1. doi: 10.1186/s12860-018-0182-z (PMC6446504; doi:10.1186/s12860-018-0182-z)

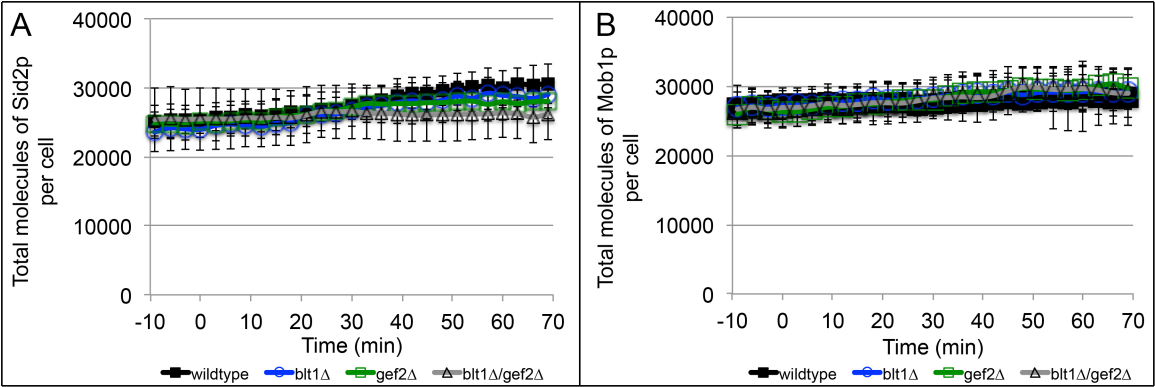

Supplement: Supplementary file 1 — Cellular concentrations of Sid2p-mEGFP and Mob1p-mEGFP are constant between wildtype and mutant strains during mitosis. (ZIP 696 kb) [file 12860_2018_182_MOESM1_ESM.zip › Additional File 1.pdf]

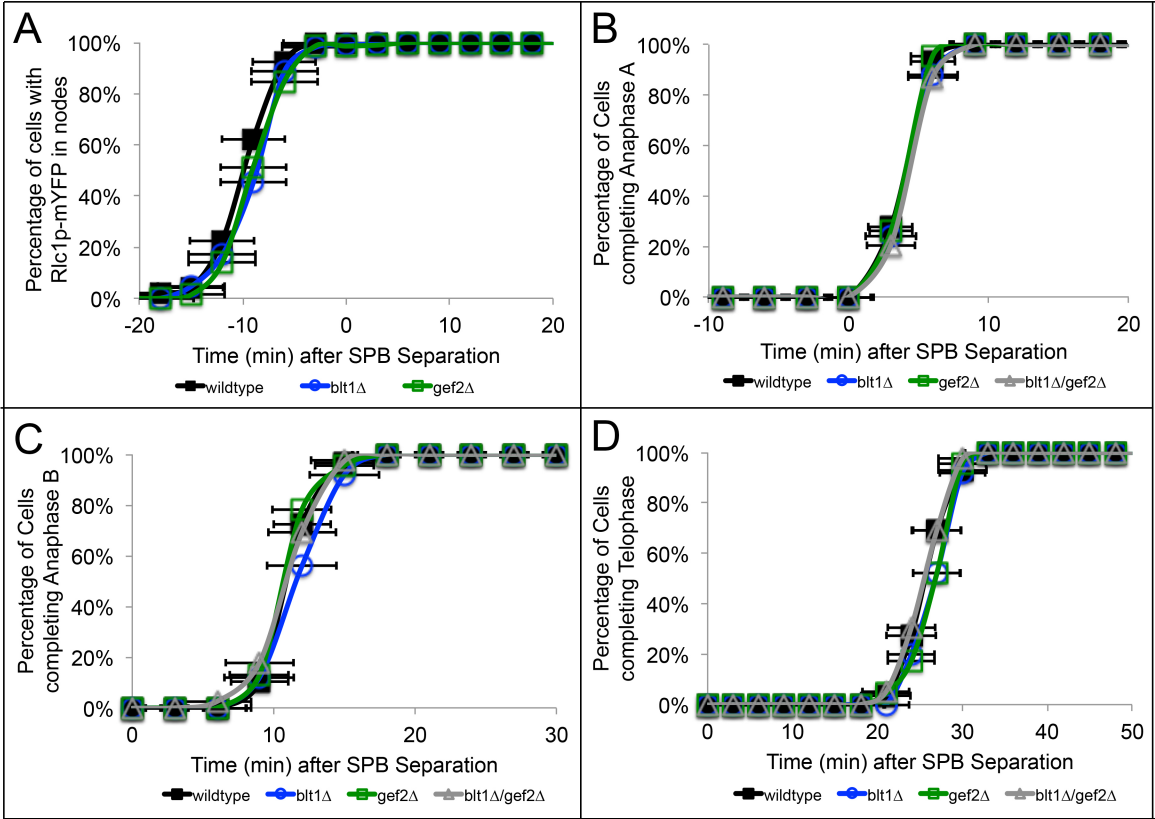

Supplement: Supplementary file 2 — Rlc1p-mYFP recruitment to nodes and initiation of anaphase and telophase are normal in blt1∆, gef2∆, and blt1∆/gef2∆ mutants. (ZIP 1277 kb) [file 12860_2018_182_MOESM2_ESM.zip › Additional File 2.pdf]

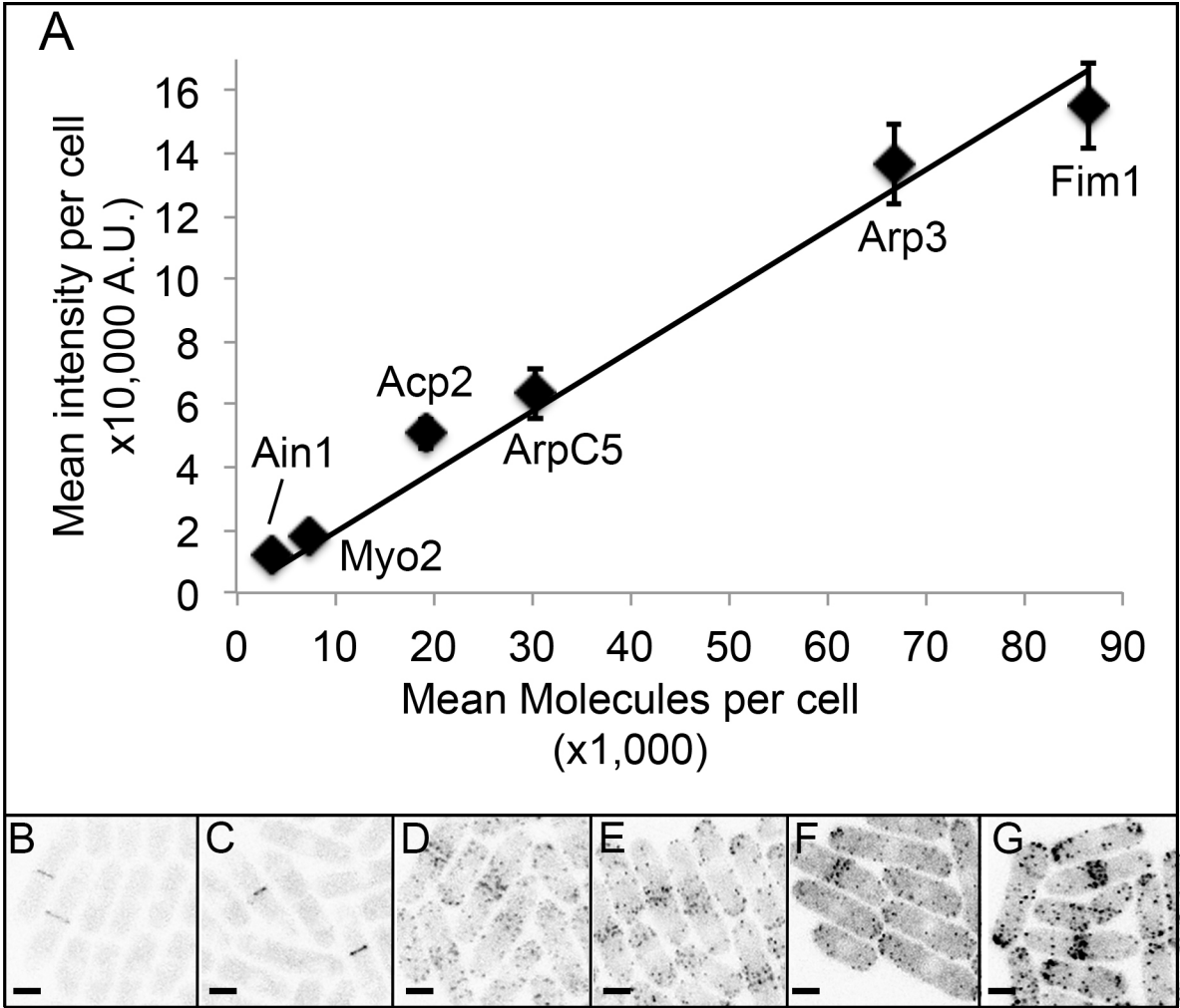

Supplement: Supplementary file 3 — Standard curve with measurement of mean molecules per cell by fluorescence microscopy. (ZIP 507 kb) [file 12860_2018_182_MOESM3_ESM.zip › Additional File 3.pdf]
