# Supplementary material for: Fission yeast type 2 node proteins Blt1p and Gef2p cooperate to ensure timely completion of cytokinesis
Source: BMC Mol Cell Biol. 2019 Jan 24;20:1. doi: 10.1186/s12860-018-0182-z (PMC6446504; doi:10.1186/s12860-018-0182-z)
Supplement: Supplementary file 2 — Rlc1p-mYFP recruitment to nodes and initiation of anaphase and telophase are normal in blt1∆, gef2∆, and blt1∆/gef2∆ mutants. (ZIP 1277 kb) [file 12860_2018_182_MOESM2_ESM.zip › Additional File 2.docx]

**Additional File 2: Rlc1p-mYFP recruitment to nodes and initiation of anaphase and telophase are normal in *blt1Δ*, *gef2Δ*, and *blt1Δ/gef2Δ* mutants.**

Time shown in minutes; time zero represents SPB separation. (A) Time course of the localization of Rlc1p-mYFP to nodes in wildtype cells (black line, ◼; n = 90), *blt1Δ* cells 19

(blue line, 🞆; n = 102), *gef2Δ* cells (green line, ☐; n = 65) or *blt1Δ*/*gef2Δ* cells (gray line, Δ; n = 118). Error bars represent ± 1 SD. Time course of (B) Anaphase A – SPB localization to opposing poles of the nuclear envelope, (C) Anaphase B – initiation of SPB movement to the cellular poles, and (D) Telophase – completion of SPB movement to the cellular poles; as observed through SPB resident protein Sad1-RFP in wildtype cells (black line, ◼; n = 29), *blt1Δ* cells (blue line, 🞆; n = 25), *gef2Δ* cells (green line, ☐; n = 23) or *blt1Δ*/*gef2Δ* cells (gray line, Δ; n = 39). Error bars represent ± 1 SD.
