# Supplementary material for: Fission yeast type 2 node proteins Blt1p and Gef2p cooperate to ensure timely completion of cytokinesis
Source: BMC Mol Cell Biol. 2019 Jan 24;20:1. doi: 10.1186/s12860-018-0182-z (PMC6446504; doi:10.1186/s12860-018-0182-z)
Supplement: Supplementary file 3 — Standard curve with measurement of mean molecules per cell by fluorescence microscopy. (ZIP 507 kb) [file 12860_2018_182_MOESM3_ESM.zip › Additional File 3.docx]

**Additional File 3: Standard curve with measurement of mean molecules per cell by fluorescence microscopy.**

(A) Representative correlation plot of mean molecules per cell [62] with mEGFP fluorescence intensity per cell (mean ± 1 SD, n ≥ 25 cells per strain; y = 1.9251x; R2 = 0.98). Error bars represent ± 1 SD. Representative images of fluorescence micrographs from two-dimensional sum intensity projections of 10 z-sections spaced at 1.0μm intervals for strains expressing integrated mEGFP fusion proteins tagging endogenous (B) Ain1, (C) Myo2, (D) Acp2, (E) ArpC5, (F) Arp3, and (G) Fim1. Scale bar = 3μm.
